# Toward Sustainable Aviation: Minimizing Aircraft Contrail Ice Particle Formation and Climate Effects by Controlled Seeding of Ice Nuclei Particles

**DOI:** 10.1021/acsestair.5c00241

**Published:** 2025-12-01

**Authors:** Fangqun Yu

**Affiliations:** Atmospheric Sciences Research Center, 1084 University at Albany, Albany, New York 12226, United States

**Keywords:** sustainable aviation, contrail avoidance, seeding of ice nuclei particles, contrail cirrus, aviation non-CO_2_ climate impact

## Abstract

Global aviation has contributed ∼3.5% to the anthropogenic climate forcing in 2018, of which around two-thirds (with substantial uncertainty) were due to non-CO_2_ effects dominated by contrail cirrus. To be sustainable, the aviation industry faces a great challenge in reducing its climate footprint. There are ongoing efforts toward contrail avoidance via rerouting flights to avoid ice supersaturated regions, but serious reservations have been voiced against it because of extra fuel burning and resultant increased CO_2_ emissions, among other issues. Based on simulations with a state-of-the-art aerosol and contrail microphysics model, we show that the aviation non-CO_2_ climate effect associated with contrail cirrus may be significantly reduced via controlled seeding of a small amount of ice-nucleating particles (INPs). The optimized amount of INPs seeded will consume water vapor and minimize the peak relative humidity reached in the plume. In turn, this reduces the number of exhaust particles activating and forming contrail ice particles by up to 1–2 orders of magnitude, resulting in larger contrail ice particles that fall faster and shorter contrail lifetimes, which is expected to diminish the warming effect of contrail cirrus to a very small level. This novel approach may solve some of the issues associated with the proposed navigational contrail avoidance, but further research is needed to assess its feasibility and environmental impacts.

## Graphical abstract

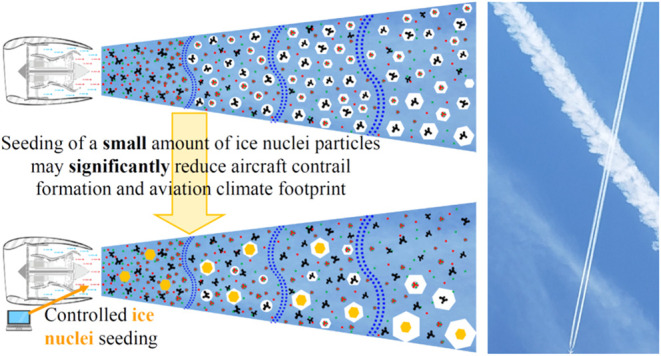

## 1 Introduction

The aviation industry faces a great challenge in reducing its climate footprint. Global aviation contributed ∼3.5% to the anthropogenic global heating in 2018,^1^ of which around one-third was caused by the greenhouse effect of emitted CO_2_ while the other two-thirds (with large uncertainty) were non-CO_2_ effects because of strong preceding growth rates of aviation.^1–3^ The climate footprint of aviation is expected to increase substantially as a result of the projected factor of ∼3.8 increase in air traffic from 2018 to 2050.^4^ In addition to decarbonization, e.g., by using sustainable aviation fuels (SAF), the aviation industry needs to address non-CO_2_ climate effects.^5^ The impact associated with contrail cirrus dominates the non-CO_2_ effects and has a net warming effect, although the uncertainty is large, and the level of confidence is low.^1,3^ The European Commission extends the Monitoring, Reporting, and Verification (MRV) framework to cover non-CO_2_ aviation effects and aircraft operators will have to monitor their non-CO_2_ effects starting January 1, 2025.^6^

It has been proposed that the contrail cirrus climate impact be mitigated through contrail avoidance by rerouting flights.^7^ A number of theoretical analyses and modeling studies indicate that navigational contrail avoidance may be beneficial.^8–10^ However, serious reservations over contrail avoidance by rerouting flights have been voiced.^3,11,12^ To explore the feasibility, EUROCONTROL and the German Aerospace Center (DLR) conducted the first operational trial on the avoidance of contrails over a period of 10 months in 2021 in the Maastricht Upper Area Control Center (MUAC).^13,14^ Delta Air Lines teamed up with the Massachusetts Institute of Technology (MIT) to study contrail avoidance in 2022.^15^ In 2023, American Airlines participated in a first-of-its-kind study led by Google Research and Breakthrough Energy to test whether atmospheric zones likely to create contrails can be identified with artificial intelligence (AI) and avoided.^16^ A small group of American Airlines pilots flew 70 flights over six months, using AI-based predictions to make small modifications to routes that were projected to create contrails. These trials, while limited in scale, provide information about the feasibility of navigational contrail avoidance, but many issues need to be addressed prior to operational implementation.

Serious reservations over navigational contrail avoidance include the large uncertainties in the actual climate effects of contrail cirrus, extra fuel burning and associated CO_2_ emissions, and various operation challenges such as the need for accurate ice supersaturated region (ISSR) prediction not only in the planned route but also in the contingency route, the necessity to carry extra fuel (and thus increase fuel weight and efficiency), airspace constraints and safety consideration, flight delays, additional operation burdens, validation difficulty, and governance issues.^3,11,12^

Here, we propose and demonstrate a novel approach that can potentially reduce the climate impact of contrail cirrus and is not affected by the above-mentioned challenges and concerns associated with navigational contrail avoidance. The approach is to minimize the number of ice particles formed in those persistent contrails having warming effects via controlled seeding of an optimized and small amount of efficient ice nuclei particles (INPs). Section 2 details the INP seeding method and its advantages as well as the model used in this study. Section 3 provides results and discussion.

## 2 Methods

### 2.1 COntrail Mitigation By Ice Nuclei Effective Seeding (COMBINES)

Contrail ice crystals form when aerosol particles (including nonvolatile soot particles, volatile particles, and entrained ambient particles) in the exhaust plume activate into droplets in water supersaturated conditions and subsequently freeze by homogeneous nucleation.^2,17–20^ Contrail ice formation is generally dominated by nonvolatile soot particles in flights with rich-burn engines^19,21–24^ but volatile particles can become important in lean-burn engines with low soot emissions,^18,20^ although there exist large knowledge gaps over factors influencing the contribution of volatile particles to contrail ice formation and the effects of fuel compositions and engine types on volatiles particle formation.

The contrail cirrus climate effect depends on the number of ice crystals formed per kg of fuel consumption (EI_ice_). Limited global simulations based on limited models suggest that a factor of 2 reduction in EI_ice_ can reduce the associated RF by ∼14–20% ^25,26^ while a factor of 5 reduction in EI_ice_ can cut the contrail cirrus RF by half.^25^ Another study suggests that a factor of ∼6 reduction in EI_ice_ can cut the RF by 35%.^27^ Apparently the warming impacts of contrail cirrus can be reduced if the number of contrail ice crystals formed can be reduced, although there exist uncertainties in the magnitude of reduction. The novel approach we propose here, as detailed below and in Section 3, can reduce EI_ice_ by a factor of up to ∼50 or more and is expected to diminish the warming effect of contrail cirrus to a very small or insignificant level.

The contrail formation processes in present aircraft jet engines are illustrated in Figure 1a. Depending on ambient conditions, fuel compositions, regular engine technologies and operation conditions, the number of ice crystals generated per kg of fuel consumption can reach up to ∼10^15^–10^16^ (i.e., ice emission index EI_ice_ = 10^15^–10^16^/kg-fuel).^20^ Because of their large numbers and limited water vapor available, ice crystals formed in the engine exhaust plume initially grow slowly, remain small in size and settling velocity, and thus can stay in the air for a long time (becoming persistent contrail or contrail cirrus).

**Figure 1:**
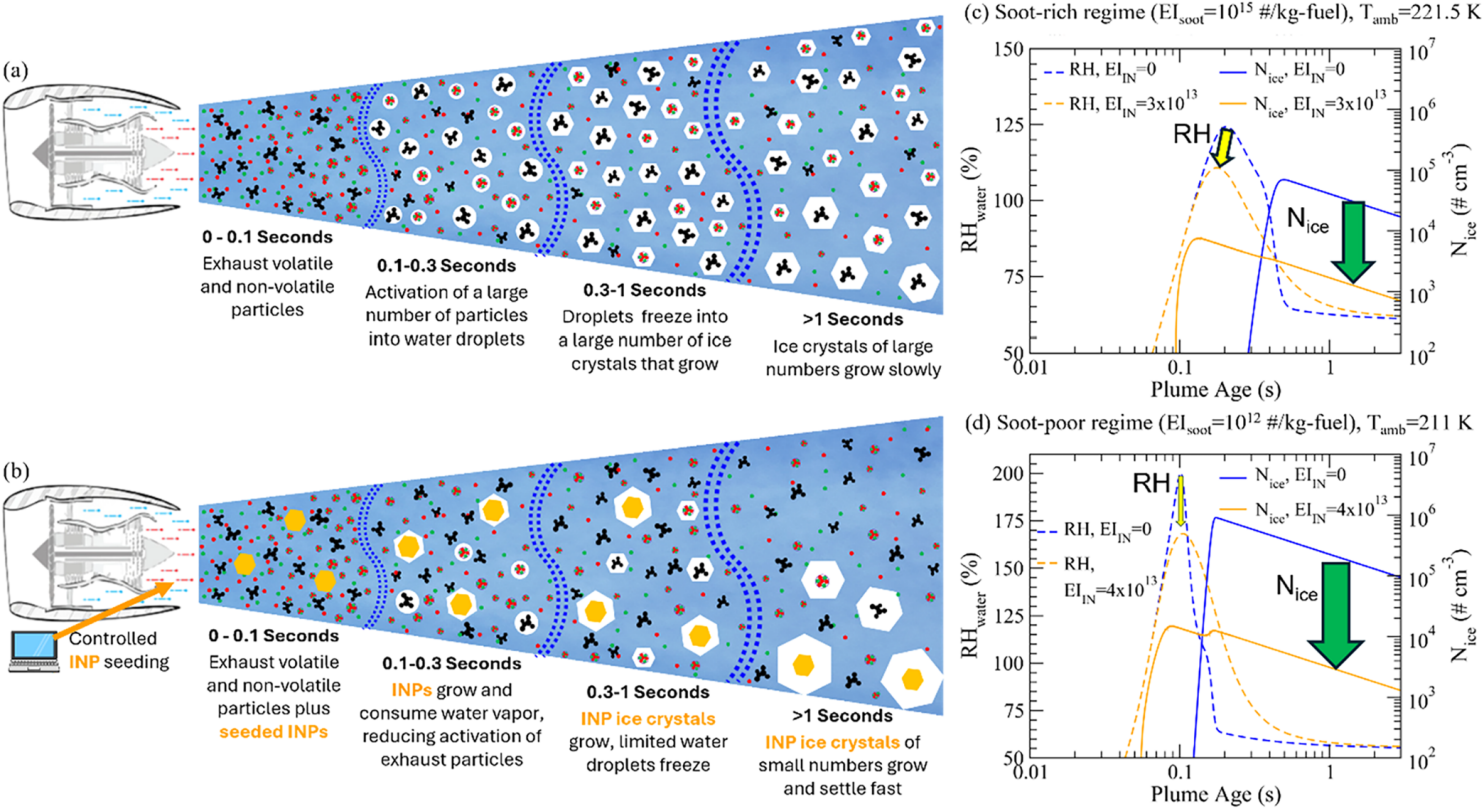
Schema of contrail formation in aircraft exhaust plumes under two scenarios: (a) baseline case for typical current aircraft engines with contrails formed on nonvolatile soot particles (black) and volatile particles composed of sulfate (red) and organics (green), and (b) proposed contrail mitigation via controlled seeding of efficient ice-nucleating particles (INPs, indicated with orange filled hexagon). (c) and (d) are model simulated impact of INP seeding (amounts indicated as EI_INP_ or EI_IN_, in #/kg-fuel) on relative humidity (RH) with respect to water (RH_water_) (indicated with yellow arrow) and number of contrail ice crystals (*N*_ice_) formed (indicated with green arrow) in aircraft plumes for two selected cases: one in soot rich regime (with soot emission index EI_soot_ = 10^15^/kg-fuel, *T*_amb_ = 221.5 K) and the other in soot-poor regime (EI_soot_ = 10^12^/kg-fuel, *T*_amb_ = 211 K). The ambient RH with respect to ice (RH_ice_) is assumed to be 110% and other relevant parameters are described in Section 2.2. Seeding the engine exhaust with a small but optimized amount of INPs can significantly decrease the number of exhaust particles forming contrail ice crystals and, thus, substantially reduce the warming effects of persistent contrails.

Figure 1b is a schematic of the proposed contrail reduction method (COMBINES). The key idea is to seed the engine exhaust with a small but optimized amount of efficient INPs when persistent contrails with a warming effect are predicted. The seeding INPs can be silver iodide (AgI) widely used in various weather modification operations^28^ or bismuth triiodide (BiI_3_) suggested for thinning cirrus clouds^29^ or other suitable materials that can freeze efficiently and have low environmental impacts. The optimal injection location depends on effectiveness and engineering feasibility for the seeding system installation and associated cost. Injection of INPs into bypass air is likely a good option but injection into core exhaust or other locations can also be considered. The temperature of core exhaust gases exiting a commercial aircraft engine’s nozzle is typically around 600 K during nominal operation. AgI and BiI_3_ have melting points of 831 and 682 K and boiling points of 1779 and 815 K, respectively. Both solid AgI and BiI_3_ are unlikely to evaporate or shatter if injected into the exhaust exiting the nozzle. If the engine exit temperature is above the melting or boiling points of injected INPs, the injection can be at a location with lower temperature to avoid melting or evaporation of injected INPs. It should be noted that water vapor is mostly produced in the core of the turbofan engine. The core exhaust has a high temperature and contrails can only form when the core exhaust cools down by mixing with bypass and ambient air. INPs, if injected into bypass air, will mix into the core exhaust before the contrail forms. The exact location of the injection to maximize effectiveness also depends on the mixing process, which remains to be evaluated. The amount of INPs seeded is to be optimized based on engine emissions and ambient conditions to minimize the total number of ice crystals formed and thus maximize their sizes and falling speed. The optimization is critical to avoid both insufficient seeding and overseeding. Further research, both numerical simulation and experimental testing, is needed to develop a suitable INP seeding system (and INP materials) and optimize the injection location/amount.

The key physics behind COMBINES, as illustrated in Figure 1c,1d, is that the seeded INP will freeze heterogeneously, take up water vapor well before RH_water_ in the plume reaches 100% (i.e., well before soot and volatile particles (including lubrication oil) are activated and can take up water vapor), substantially reduce maximum RH_water_ reached in the exhaust plume and inhibit the formation of contrail ice crystals from nonvolatile and volatile particles. Figure 1c,1d, simulated with the aerosol and contrail microphysics (ACM) model described in Section 2.2, show the impact of INP seeding on evolution of RH_water_ and number of contrail ice crystals (*N*_ice_) formed in aircraft plumes for two selected cases: one for soot rich regime (Figure 1c) and the other for soot-poor regime (Figure 1d). As a result of heterogeneous freezing, *N*_ice_ begins to increase before the plume reaches water supersaturation in the cases with seeded INPs (orange solid lines), much earlier than those without seeding (blue solid lines) (Figure 1c,1d). The ice particles resulting from seeded INPs have more time to consume water vapor and have an advantage in competing for the water vapor with liquid droplets formed via the activation of plume particles (when RH_water_ > 100%) because of much lower saturation vapor pressure over ice. The uptake of water vapor by seeded INPs reduces the maximum RH_water_ reached in the plume (indicated by yellow arrows in Figure 1c,1d) and thus decreases the number of plume particles activated into liquid droplets and later frozen into contrail ice crystals (indicated with green arrows). Under the conditions given for the soot-rich case, seeding of INPs at an amount of 3 × 10^13^/kg-fuel reduces the peak RH_water_ by ∼ 14% and *N*_ice_ by a factor of 24 (Figure 1c). For the soot-poor case, seeding of INPs at an amount of 4 × 10^13^/kg-fuel reduce the peak RH_water_ by ∼32% and N_ice_ by a factor of 73 (Figure 1d). After RH_water_ reaches its maximum value in the plume, the rate of its decrease is slower in the case of seeded INPs because of lower *N*_ice_ and thus surface areas of ice particles.

Table 1 summarizes the advantages and challenges of the proposed COMBINES approach when compared to the contrail avoidance by rerouting. Since COMBINES does not need rerouting and thus may solve some of the key challenges and concerns associated with navigational contrail avoidance, as listed in Table 1. For COMBINES operation, the amount of INP material consumed per flight is small (see Section 3), similar to the engine lubrication oil consumption rate, and should thus be easy to carry. The main challenge of COMBINES is the need to develop a suitable low-weight INP seeding system and the associated cost for installation and maintenance. If an effective low-weight INP seeding system (including the generation and storage of INP materials) can be developed, the proposed COMBINES approach can be effective for contrail mitigation under almost all situations, including various engine technologies (i.e., both rich-burn and lean-burn), aviation fuel types (jet-A and SAF), fuel compositions (fuel sulfur, aromatic, and hydrogen contents), and engine lubrication oil venting methods.

**Table 1:** Advantages and Challenges of COntrail Mitigation by Ice Nuclei Effective Seeding (COMBINES) Compared to Contrail Avoidance by Rerouting

navigational contrail avoidance	COMBINES
(1) Extra fuel burning and CO_2_ emissions.	No extra fuel burning and CO_2_ emissions but a small amount of INP materials will be injected into the upper troposphere.
(2) Need accurate ISSR prediction not only in the planned route but also in the rerouted route.	Need accurate ISSR prediction only in the planned routes which can be assessed/validated with RH sensors on planes.
(3) Planes need to carry extra fuel for potential contrail avoidance, which affects fuel efficiency.	The weight of seeding materials is negligible. The seeding system remains to be developed, and the impact of injection system weight is to be assessed.
(4) Rerouting may cause overcrowding of airspace, which may cause safety concerns.	No change to airspace and no safety concerns.
(5) Not all warming contrails can be avoided due to airspace complexity. As a result, the overall effectiveness is limited and it requires priority decisions on flights to be rerouted.	Can operate on all flights needed. No such limitation and additional decision burden.
(6) Reroute may need flights to deviate from optimized paths and thus may increase flight time and cost and lead to delay.	No need to reroute and thus no additional time and cost and no delays.
(7) Additional burden for flight operation personnel and associated costs.	INP seeding operation can be automated after a suitable seeding system is developed and installed.
(8) Rerouting does not change the ISSR and the avoided contrails may reduce dehydration and thus increase the duration and spatial coverage of ISSRs which may in turn require more rerouting or increase the formation of warming natural cirrus clouds. (Highly uncertain, research needed)	Will dehydrate more effectively (than regular unseeded contrails) and reduce ISSR duration and spatial coverage. May also reduce the formation of warming natural cirrus clouds (i.e., double benefits). (Highly uncertain, research needed)
(9) Hard to verify because it requires validation that the route avoided is indeed ISSR while the new routine is not ISSR.	Only need to validate that contrails formed are shorter-lived or have lower optical depth.
(10) Requiring methods to quantify the trade-off between CO_2_ and non-CO_2_ climate impacts of targeted flights.	Not needed and thus operationally conceptually simpler and efficient but need to assess INP seeding system CO_2_ penalty on a life-cycle basis.
(11) Cost: can be substantial because of extra fuel consumption and operation burden.	Development/testing, installation, and maintenance cost of the INP seeding system can be substantial. Need to assess the plausibility of retro-fitting the existing fleet.

### 2.2 Aerosol and Contrail Microphysics (ACM) Model

A state-of-the-art ACM plume model, as detailed in Yu et al.,^20^ is employed for this study. The ACM model is a parcel model of jet plume aerosol and ice microphysics developed in the late 1990s,^17,30,31^ with the volatile particle formation portion improved with algorithms and thermodynamic data developed in the past two decades^32^ and the contrail microphysics portion improved with a new soot activation scheme.^20^ The ACM model captures the dependence of contrail ice particles formed on emission indices of nonvolatile soot particles and can explain less-than-unity fractions of soot particles forming contrail ice particles^20^ as recently observed during ECLIF (Emission and CLimate Impact of alternative Fuels) campaigns.^33,34^ More importantly, the ACM model simulations reveal that, because of the activation of volatile particles, the number of contrail ice particles formed when soot emission is very low (i.e., in the soot-poor regime) can be comparable to that of the soot-rich regime.^2,18,20^

To study the impact of INP seeding on the formation of contrail ice crystals under a variety of conditions, we add one additional type of particles representing seeded INPs into the ACM model. In this study, the seed INPs are assumed to be AgI and have a log-normal distribution with a median diameter of 100 nm and a standard deviation of 1.3. The heterogeneous freezing rates of INPs (as a function of temperature) are derived from laboratory measurements.^28,35^ The contrail ice crystals formed on these INPs are tracked separately from those formed on soot, volatile, and entrained ambient particles.

Same as in the ECLIF studies,^20^ the average dilution ratio as a function of plume age is based on the parametrization derived by Schumann et al.,^36^ and ambient particles entrained into the plume is assumed to have two log-normal modes with number concentration, dry median diameter, and standard deviation of 1000 #/cm^3^, 10 nm, 1.6 and 10 #/cm^3^, 150 nm, 1.6, respectively. The size distribution of soot particles (aggregates), based on ECLIF ground-based characterization of an Airbus A320,^37^ is assumed to be log-normal with a median diameter of 35 nm and a standard deviation of 1.6. The ratio of the diameter of primary soot particles to that of aggregates is assumed to be 0.41.^20^ The present study focuses on persistent contrails and the ambient relative humidity with respect to ice (RH_ice_) is fixed to be 110%, close to the median values observed during ECLIF. The conclusions of this work are not sensitive to RH_ice_.

For the simulation of volatile particles, we assume initial (at the engine exit) chemi-ion concentrations of 10^9^ #/cm^3^, and use the emission index of condensable gases (EI_GCON_) to specify the amount of compounds (sulfuric acid and organic species) that contribute to volatile formation and growth. The current jet fuel has an average fuel sulfur content (FSC) of 500 ppmm, which will contribute 50 mg/kg-fuel of sulfuric acid to EI_GCON_ if 3% of fuel sulfur is converted to sulfuric acid.^20^ EI_GCON_ due to organics was estimated to be ∼ 20 mg/kg-fuel in a previous study^31^ and may be higher based on a recent study.^38^ Considering that FSC in future aviation fuels is likely much lower than the current level, we use EI_GCON_ of 30 mg/kg-fuel as the baseline value and conduct sensitivity studies to explore its impact. The ACM model uses variable time step (dt) to ensure accuracy and computational efficiency,^20^ with dt as small as 10^–5^ s (when needed) to enable the detailed microphysics (including volatile particle formation and growth, dilution and RH_water_ peaks, and contrail formation) to be well resolved. We run the ACM model to the plume age of 150 s and the emission indices of ice (EI_ice_) at this plume age are compared.

## 3 Results and Discussion

The number of ice crystals formed in contrails depends not only on engine particle emissions but also on the maximum RH_water_ (RH_water_max_) reached in the plume. The strategy of COMBINES is to reduce RH_water_max_ and thus the number of ice crystals formed in contrails through controlled seeding of efficient INP (Figure 1). Figure 2 shows reduction levels of contrail ice formed (EI_ice_) by controlled INP seeding as a function of engine soot emission (EI_soot_) at four different ambient temperatures representative of cruise altitude conditions. It can be seen from Figure 2 that the controlled seeding can reduce the number of contrail ice particles formed by up to a factor of ∼50 (comparing solid lines with dashed or dot-dashed lines), both in soot-rich (>∼10^14^/kg-fuel, see green and orange lines) and soot-poor regimes (<∼10^14^/kg-fuel, see blue and red lines). It should be noted that the level of reduction at various EI_soot_ (and *T*_amb_) as shown in Figure 2 depends on the seeding INP emission index, EI_IN_ (discussed below) and thus is not optimized for all EI_soot_. It is interesting to note that the line at 225 K with EI_IN_ = 0/kg-fuel reaches a peak EI_ice_ at the EI_soot_ of ∼10^15^ /kg-fuel. At this temperature, as EI_soot_ increases, EI_ice_ first increases as more soot particles form droplets and then freeze. When EI_soot_ further increases to above ∼10^15^ /kg-fuel, the amount of water on each liquid droplet formed on soot particles is smaller which reduces the fraction of these droplets freezing into ice particles. Due to the dependence of freezing rate on water volume (or size of liquid droplets) and the competition of water vapor between liquid droplets and frozen ice particles, EI_ice_ peaks at EI_soot_ of ∼10^15^ /kg-fuel at *T*_amb_ = 225 K. The freezing rate is very sensitive to temperature, and this phenomenon generally occurs at higher ambient temperatures.

**Figure 2:**
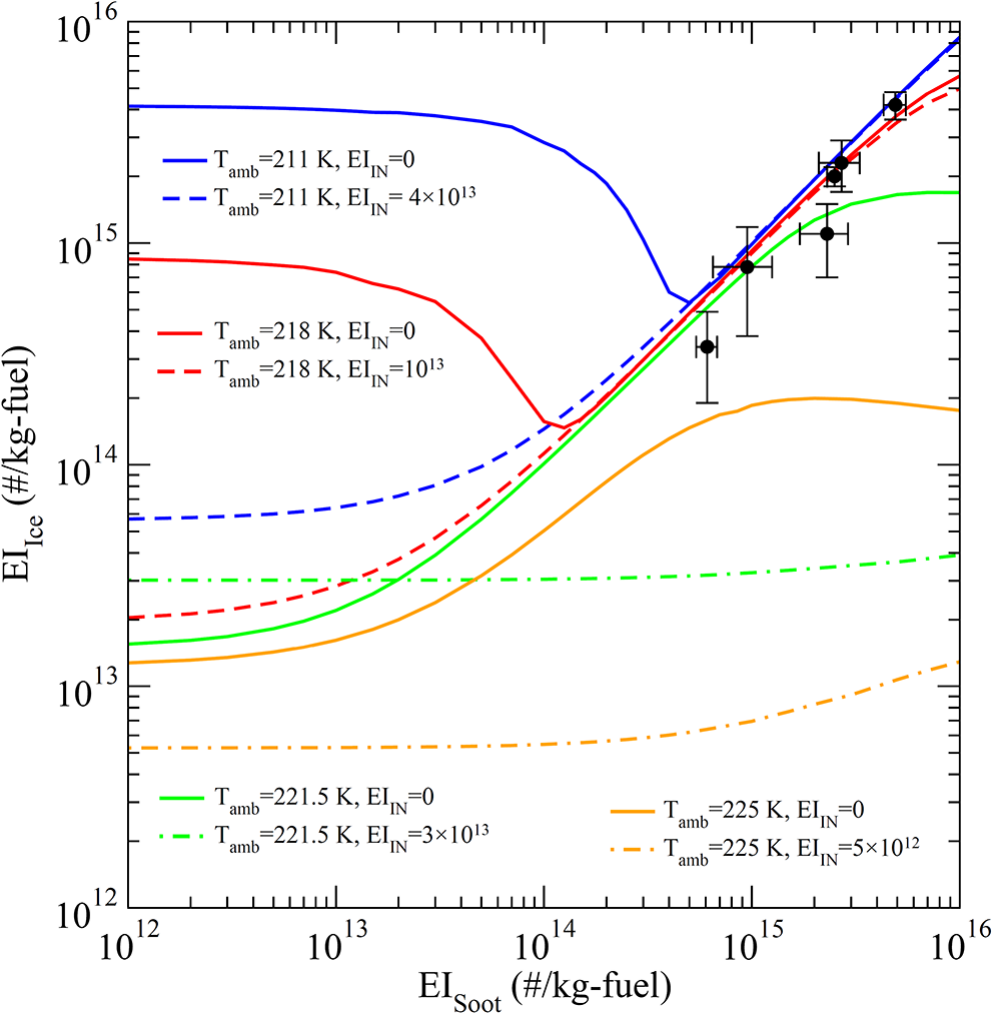
Levels of contrail ice formed (EI_ice_) reduction by controlled IN seeding as a function of engine soot emission (EI_soot_) at four different temperatures. Solid lines: no IN seeding; Dashed lines: INP seeding with seeding amount or rate (EI_IN_, in #/kg-fuel) indicated. The emission index of condensable gases (sulfuric acid and organic species) (EI_GCON_) forming volatile plume particles is assumed to be 30 mg/kg-fuel. The symbols are measurements from ECLIF (Emission and CLimate Impact of alternative Fuels) Campaigns 1–3 with ambient temperatures ranging from 213 to 220 K.^20,33,34^

The magnitudes of EI_ice_ reductions depend mainly on concentrations of soot and volatile particles, *T*_amb_, and the amount of INPs seeded. Under a given condition, the amount of INPs to be seeded can be optimized to achieve a minimum (total) number of ice crystals formed. Figure 3 shows how different amounts of seeded INs (EI_IN_) affect the EI_ice_ as a function of *T*_amb_ in soot-poor and soot-rich regimes. Seeded INs can effectively reduce EI_ice_ (by a factor of up to ∼80) when *T*_amb_ is more than ∼5 K below the contrail formation threshold *T* (*T*_th_, ∼226 K for the cases shown in Figure 3) in the soot-poor regime (i.e., *T*_amb_ < ∼221 K, Figure 3a) and when *T*_amb_ is within ∼10 K below *T*_th_ in the soot-rich regime (*T*_amb_ > ∼216 K, Figure 3b) but the amount of EI_IN_ seeded needs to be optimized to achieve maximum reduction. In the soot-poor regime and when *T*_amb_ > ∼221 K, EI_ice_ is dominated by entrained ambient particles and is already quite low. In such situations, no seeding is necessary, and seeding is not very effective or even counter-effective (i.e., increases EI_ice_ due to overseeding). In the soot-rich regime and when *T*_amb_ < ∼216 K, a higher INP amount is needed to be effective, and the magnitude of reduction in EI_ice_ is limited (down to a factor of 3 or smaller).

**Figure 3:**
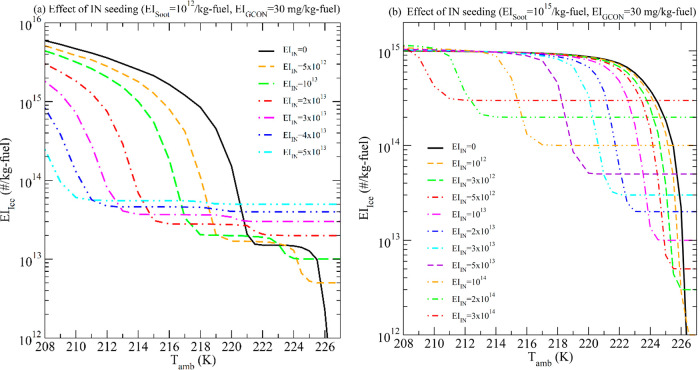
Levels of contrail ice formed (EI_ice_) reduction by controlled IN seeding as a function of ambient temperatures at different IN seeding (EI_IN_, in #/kg-fuel) in two soot emission regimes: (a) soot-poor (EI_soot_ = 10^12^/kg-fuel), and (b) soot-rich (EI_soot_ = 10^15^/kg-fuel). EI_GCON_ is assumed to be 30 mg/kg-fuel.

Due to the need to decarbonize and improve air quality, using SAF and new engine technologies (such as lean burn) is expected to significantly reduce aircraft emissions of nonvolatile soot particles. As a result, the soot-poor regime will become more dominant, and EI_ice_ is modeled to be determined by the contribution of volatile particles.^20^ The contribution of volatile particles to EI_ice_ is likely sensitive to the amount of condensable gaseous species (EI_GCON_, including sulfuric acid, organics, nitric acid, etc.) and *T*_amb_.^20^ Figure 4 shows the sensitivity of IN seeding effectiveness in reducing EI_ice_ as a function of EI_GCON_ under two *T*_amb_ (211 and 218 K). Seeding of IN at a level of 5 × 10^12^–5 × 10^13^/kg-fuel can reduce EI_ice_ by a factor of up to ∼100. The level of IN seeded to minimize EI_ice_ depends on EI_GCON_ as well as *T*_amb_.

**Figure 4:**
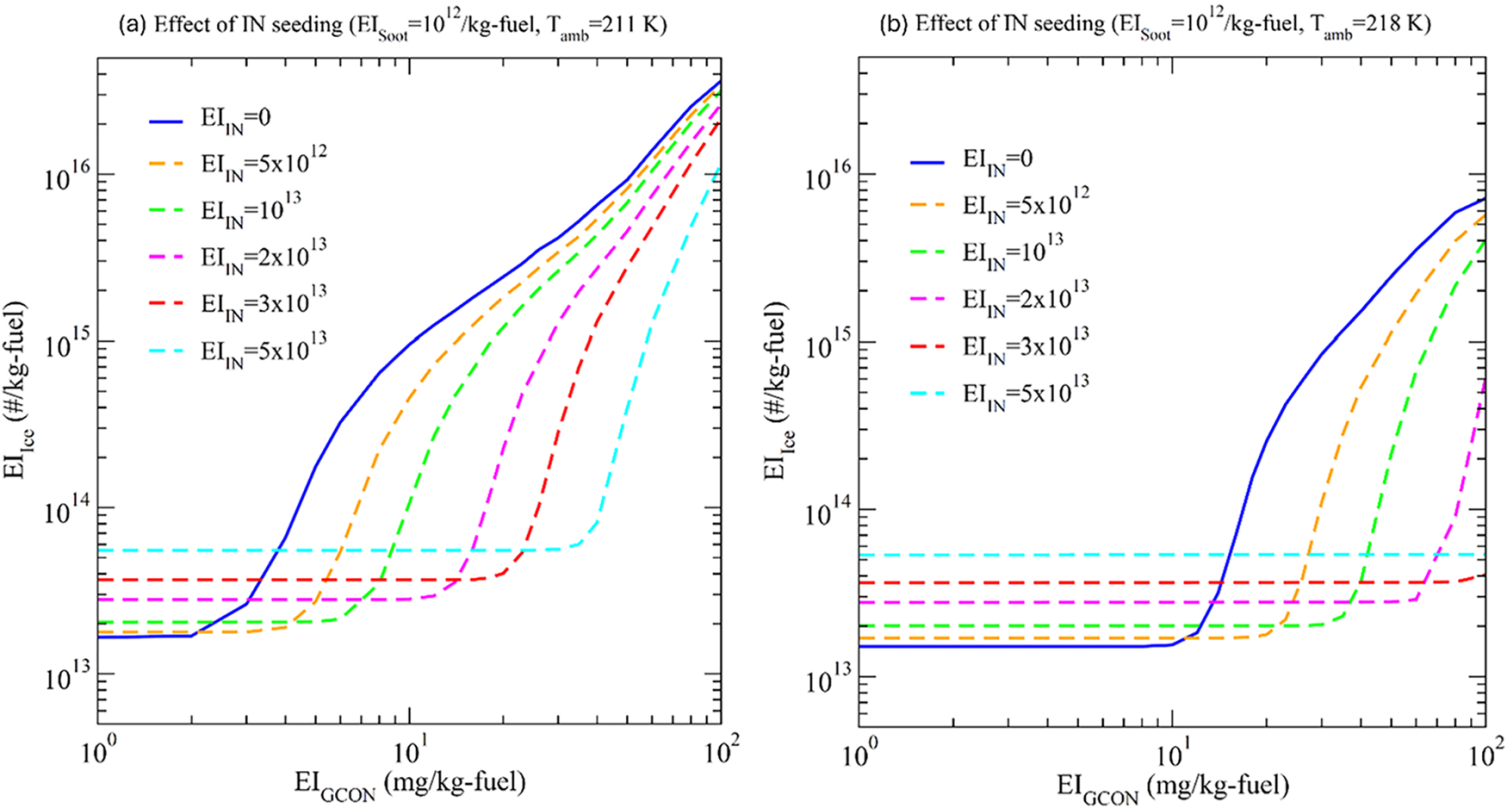
Levels of contrail ice formed (EI_ice_) reduction by controlled IN seeding as a function of EI_GCON_ at different IN seeding (EI_IN_, in #/kg-fuel) in soot-poor regime (EI_soot_ = 10^12^/kg-fuel) for two ambient temperatures (a) *T*_amb_ = 211 K, and (b) *T*_amb_ = 218 K.

It can also be seen from Figure 4 that the lower the EI_GCON_ the smaller the EI_ice_. The contribution of volatile particles to EI_ice_ becomes negligible when EI_GCON_ is below a certain level, ∼2 mg/kg-fuel at *T*_amb_ = 211 K and ∼10 mg/kg-fuel at *T*_amb_ = 218 K. The EI_GCON_ of current engines and fuels is likely to be well above 10 mg/kg-fuel. While sulfur in the fuel can be reduced to a very low level (<5 ppmm), it will be challenging to reduce unburned organic compounds in engines running on kerosene or SAF to a very low level. In addition, it has been recently shown that lubrication oil particles may potentially contribute to contrail ice formation for kerosene and hydrogen combustion.^39^ Assuming the emission of lubrication oil into the exhaust plume can be avoided, we expect very low emissions of condensable sulfur and organic species in future aircraft running on hydrogen fuel but nitric acid and other precursor gases from ambient air are unavoidable. It should be noted that the results shown in Figure 4 are based on the water emission level of jet fuel or SAF. The water emission index for hydrogen fuel will be a factor of ∼2.6 higher and EI_GCON_ needed to inhibit the contribution of volatile particles to EI_ice_ will be much lower. While the feasibility, cost, and time needed to reduce EI_GCON_ to a level that can inhibit the contribution of volatile particles to EI_ice_ remain to be studied, the INP seeding approach proposed here can be effective in reducing EI_ice_ and the warming effects of contrail cirrus.

One unique feature of the COMBINES approach is that EI_IN_ can be optimized to minimize EI_ice_. To illustrate this further, Figure 5 presents EI_ice_ (a,b) and corresponding mass-weighted mean diameter of ice crystals (*D*_ice_, c,d) at plume age of 150 s as a function of EI_INP_ at different ambient temperatures in two soot emission regimes for two conditions. Under the conditions shown in Figure 5a for the soot-poor regime, the optimized EI_INP_ ranges from 5 × 10^12^/kg-fuel at *T* = 220 K to 7 × 10^13^/kg-fuel at *T* = 209 K. Under the conditions shown in Figure 5b for the soot-rich regime, the optimized EI_INP_ ranges from 5 × 10^12^/kg-fuel at *T* = 225 K to 5 × 10^14^/kg-fuel at *T* = 209 K. As expected, *D*_ice_ increases with decreasing EI_ice_ because the amount of water vapor available for growing ice particles is limited. Previous model simulations indicated a reduction of contrail lifetime and optical depth when initial ice number or EI_ice_ is reduced^40,41^ and a longer lifetime of contrail-cirrus compared to natural cirrus due to high number concentrations and smaller sizes of ice particles in contrail-cirrus.^42^ The seeding of INPs can effectively reduce EI_ice_ and increase *D*_ice_, resulting in a weaker climate impact of contrail cirrus. Assuming the seeding material to be bismuth triiodide (BiI_3_) or silver iodide (AgI) and INP diameter of 100 nm, EI_INP_ of 5 × 10^12^–5 × 10^14^/kg-fuel corresponds to mass consumption of ∼15–1500 mg/kg-fuel. Since only a fraction of the flight distance needs seeding, the consumption rate of seeding materials is similar to that of lubrication oil. It should be noted that the INP mass needed can be decreased by a factor of 8 if an INP diameter of 50 nm is used. Laboratory measurements indicate a similar ice freezing efficiency of AgI in the size range of 40–400 nm.^28^ Of course, the INP injection system and the technology to optimize INP seeding amounts for desired COMBINES remain to be developed. The weight penalty for carrying INP materials is likely small but that for the injection system will depend on the system to be developed. If the seeding system can be developed as an add-on component, it can be installed only when needed (i.e., not installed all the time).

**Figure 5:**
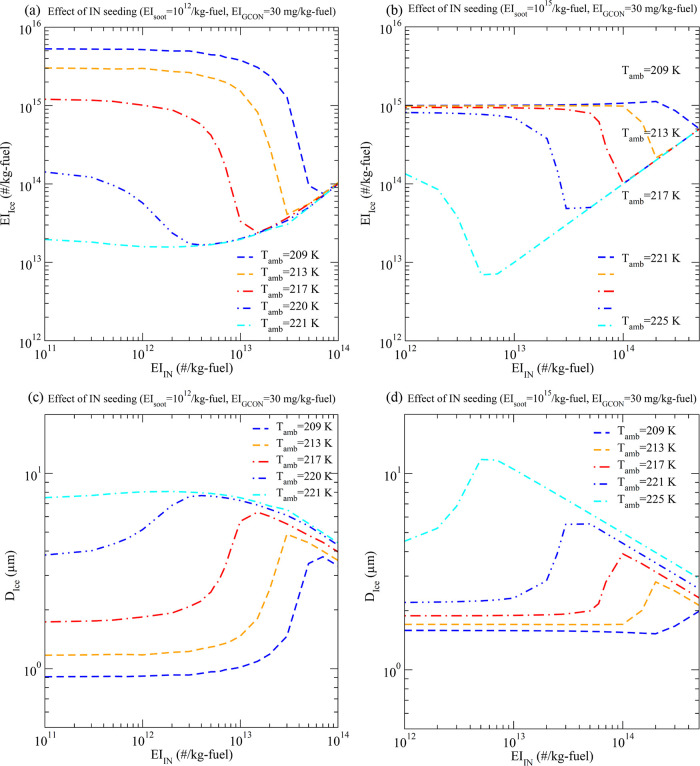
Emission index (EI_ice_, a, b) and mass-weighted mean diameter (*D*_ice_, c, d) of contrail ice formed by controlled IN seeding as a function of EI_IN_ at different ambient temperatures (*T*_amb_) in two soot emission regimes: (a, c) soot-poor (EI_soot_ = 10^12^/kg-fuel), and (b, d) soot-rich (EI_soot_ = 10^15^/kg-fuel). EI_GCON_ is assumed to be 30 mg/kg-fuel.

To achieve a maximum reduction in contrail cirrus climate effects, the amount of INPs to be seeded needs to be optimized according to engine emissions (which depend on fuel composition and engine operation information) and ambient air conditions. The ACM model used for the present study, while representing state-of-the-art as a microphysical plume model, is subject to uncertainties associated with particle thermodynamics and microphysics, parametrization of plume dilution, and model inputs. One known uncertainty is associated with inhomogeneous mixing in the exhaust plume which has been shown to affect contrail ice crystal formation and survival.^43,44^ Via comparison of contrail ice formation based on 3D large-eddy simulations (LES) compared to those in a 0D box model,^43^ it has been shown that the activation of soot is largely overestimated in plume box model approaches. Another uncertainty is related to the size of primary soot particles.^20^ Sensitivity studies have been carried out to assess the uncertainties associated with these two parameters (Figure 6). It can be seen that a faster mixing reduces the maximum EI_ice_ reduction with INP seeding but the magnitude of reduction is still substantial (by more than a factor of 10) in the fast mixing scenario. Regarding the effect of the size of primary soot particles or the ratio of the size of primary soot particles (*d*_p_) to that of aggregates (*D*_p_), there is not much difference in EI_ice_ versus EI_IN_ between *d*_p_/*D*_p_ = 0.41 and 1.0 but a very small *d*_p_ (*d*_p_/*D*_p_ = 0.2) reduces the magnitude of EI_ice_ decrease associated INP seeding.

**Figure 6:**
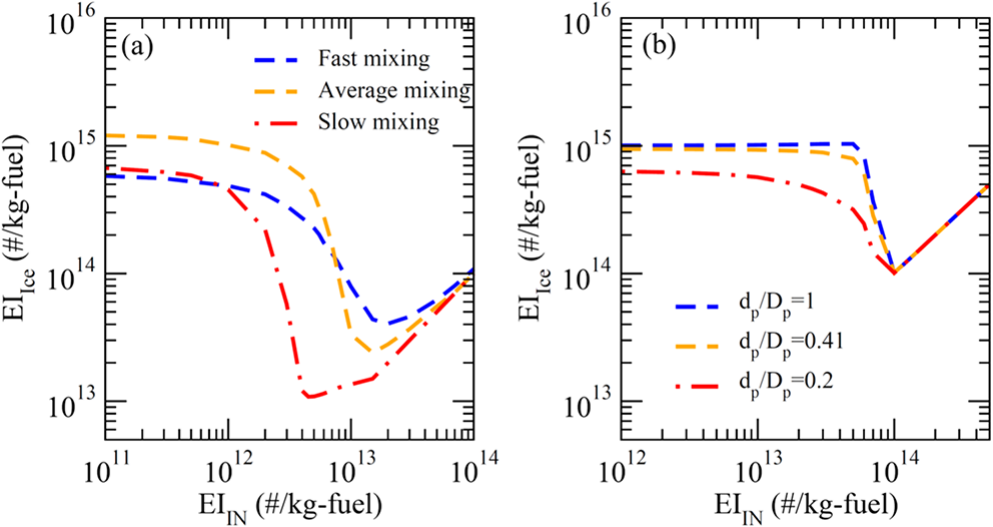
Same as Figure 5 but looking into the effects of (a) plume mixing rates (fast, average, and slow mixing rates as defined in^32^), and (b) ratios of the size of primary soot particles (*d*_p_) to that of aggregates (*D*_p_) at *T*_amb_ = 217 K. (a) is for soot-poor (EI_soot_ = 10^12^/kg-fuel), and (b) is for soot-rich (EI_soot_ = 10^15^/kg-fuel). EI_GCON_ is assumed to be 30 mg/kg-fuel for both cases.

Despite various uncertainties, the key conclusion of this work, i.e., optimized seeding of efficient INP can reduce contrail ice formation and potentially, climate impact, is robust. While the exact amount of INPs to be seeded optimally under a given condition may vary, we do not expect it to differ significantly from the values presented in this study. Laboratory experiments and field measurements are needed to validate model prediction, reduce model uncertainties, and refine the COMBINES approach detailed in this study, enabling more accurate optimization calculations under various conditions (including engines running on SAF and hydrogen fuels). 3D LES simulations can help to evaluate the limitations of box model approaches used in present study and implications of inhomogeneous mixing in jet exhaust plumes to the optimal EI_IN_ values under various conditions.

Engineering technology to seed INPs at the optimized amount for COMBINES can build upon some of the understanding accumulated and technologies developed in the past regarding cloud seeding.^45^ The cost-effectiveness of using the proposed COMBINES for contrail mitigation depends on the development of a robust INP seeding system that is lightweight and can be automated to minimize the installation, operation, and maintenance costs. As pointed out earlier, the absolute amount of INPs to be injected to minimize the contrail warming effect is likely to be small because injection is needed only when persistent contrails with warming effects are predicted. A review of existing studies suggests silver iodide at current levels induced by the ongoing cloud seeding does not pose an environmental or health concern although it is not known whether more widespread use of the material would have an impact on the environment or public health.^45^ While the optimized injection amount of INP materials for COMBINES is small (comparable to the fleet-wide lubrication oil consumption rate), it is unknown whether the potential use of silver iodide or other INP materials in the upper troposphere (i.e., not near airports or in the lower troposphere) would be a concern for public health or the environment. The fate of the residual INP resulting from COMBINES and their impacts on ambient water vapor, natural cirrus and other clouds, local air quality, and water quality need to be evaluated.

In addition to the navigational contrail avoidance described in the Introduction, other suggested contrail reduction methods include the usage of biobased or synthetic fuels,^23,33^ the development of engine and aircraft technology,^46^ and the hydroprocessing of fossil fuel-based aviation kerosene.^47^ COMBINES proposed here provides an additional method to significantly reduce contrail ice numbers (by up to 1–2 orders of magnitude) and climate impact under various engine operation settings (e.g., rich-burn versus lean-burn), fuel types and compositions, and ambient conditions. It should be emphasized that all above contrail reduction methods are based on the arguments that the warming effect of contrail cirrus is substantial, which are based on limited global model simulations and subject to large uncertainties.^1,3,48^ It is critical to reduce the uncertainties in contrail cirrus radiative forcing assessment so a robust decision on necessity and optimum approach­(es) of contrail mitigation can be made.
